# The furosemide stress test to predict renal function after continuous renal replacement therapy

**DOI:** 10.1186/cc13871

**Published:** 2014-05-14

**Authors:** Peter HJ van der Voort, E Christiaan Boerma, Peter Pickkers

**Affiliations:** 1 Department of Intensive Care Medicine, OLVG, Oosterpark 9, 1091AC Amsterdam, the Netherlands; 2 Department of Intensive Care Medicine, Medical Center Leeuwarden, Henri Dunantweg 2, 8934AD Leeuwarden, the Netherlands; 3 Department of Intensive Care Medicine, Radboudum, Radboud University Medical Centre, P.O. 9101, 6500HB Nijmegen, the Netherlands

## 

We read with interest the recent article by Dr Chawla and colleagues showing that a furosemide stress test was able to predict the development of renal damage stage III according to the Acute Kidney Injury Network classification in critically ill patients [1]. In this study, 25 patients that subsequently developed acute kidney injury (AKI) had lower urine output 2 hours following administration of furosemide compared with 52 patients that did not develop AKI. Apart from predicting which patients will develop AKI based on the renal response to furosemide, this concept may possibly also be used to predict successful recovery of renal function after continuous renal replacement therapy (CRRT) in critically ill patients recovering from AKI.

The current practice to discontinue CRRT mostly considers increases in urine output or a fall in serum creatinine while on a constant dose of continuous renal support. Observational studies have shown that the most significant predictor for successful termination of CRRT is indeed urinary production [2]. Urinary output >400 ml/day has an area under the receiver operator curve (AUROC) of 0.81, resulting in correct classification for 79% of the patients [3]. Not surprisingly, this predictive ability was negatively influenced by the use of diuretics; however, the renal response on diuretics by itself was not considered a potential predictor.

Previously, we demonstrated that administration of furosemide compared with placebo after termination of CRRT did not improve renal function or shorten renal failure [4]. In our study, urinary production was measured over a 4-hour episode after termination of CRRT without any treatment, and was measured again 24 hours later following either continuous furosemide or placebo administration. Eighteen of the 71 included patients showed immediate recovery of renal function in this 4-hour episode, defined as a calculated endogenous creatinine clearance >30 ml/minute, and did not require continuation of CRRT. These 18 patients had a significantly higher urinary production (median (interquartile range)) than the others: 103 (78 to 208) ml versus 47 (17 to 85) ml, *P* = 0.002. The urinary production in the 4-hour period without study medication was associated with renal recovery during the hospital stay (7 (2 to 43) ml vs. 76 (45 to 130) ml, *P* = 0.001) with an AUROC of 0.79.

After this 4-hour period, 25 patients received furosemide 0.5 mg/kg/hour intravenously and 24 hours later a 4-hour urine portion was again collected. This second portion also showed a significantly higher urine production (654 (333 to 1,155) ml) in those patients in whom renal recovery occurred eventually during their hospital stay compared with those who did not recover (48 (15 to 207) ml, *P* = 0.007), resulting in an AUROC of 0.84.

In summary, we confirm that the spontaneous diuretic response after CRRT predicts the necessity for continued CRRT and in-hospital renal recovery. Moreover, the furosemide-induced diuretic response in patients without immediate recovery of renal function within 24 hours after cessation of CRRT is of additional value to predict eventual renal recovery during their hospital stay.

Authors’ response

Lakhmir S Chawla

We were delighted to read the report by van der Voort and colleagues [4]. In our study assessing the furosemide stress test, we hypothesized that the tubular handling of furosemide makes it an ideal agent to test renal tubular integrity and reserve [1]. As an organic acid, furosemide is tightly bound to serum proteins and gains access to the tubular lumen – not through filtration, but by active secretion via the human organic anion transporter system in the proximal convoluted tubule [5,6]. Once in the tubular lumen, furosemide inhibits luminal active chloride transport throughout the thick ascending limb of Henle [7]. To prevent sodium reabsorption and to increase urine flow, furosemide thus requires two distinct tubular nephron segments to be functioning – making it the perfect physiologic and clinical tool for tubular interrogation. Furosemide is therefore well suited to distinguishing progression in the setting of early AKI as well as renal recovery in the setting of previously established AKI.

We demonstrated excellent clinical performance in detecting progressive AKI with an AUROC of 0.87. In their work, van der Voort and colleagues demonstrated similar performance of the furosemide stress test with an AUROC of 0.84 for assessing renal recovery [4]. While the authors did not show the performance of specific cutoff values of urine output for the 4-hour collection after a 24-hour infusion of furosemide, they did provide interquartile ranges for each group. A cutoff value of about 300 ml appears to produce a test with very good prognostic performance for the prediction of renal recovery. This serves as a proof of concept that the furosemide stress test has potential utility across the AKI spectrum.

## Abbreviations

AKI: acute kidney injury; AUROC: area under the receiver operator curve; CRRT: continuous renal replacement therapy.

## Competing interests

The authors declare that they have no competing interests.
